# Crystal structure of di-μ-isobutyrato-κ^4^ *O*:*O*′-bis­[*cis*-di­chlorido­(dimethyl sulfoxide-κ*S*)rhenium(III)]

**DOI:** 10.1107/S2056989015017429

**Published:** 2015-09-26

**Authors:** Alexander A. Golichenko, Alexander V. Shtemenko

**Affiliations:** a Department of Inorganic Chemistry, Ukrainian State University of Chemical Technology, Gagarin Ave. 8, Dnipropetrovsk 49005, Ukraine

**Keywords:** crystal structure, rhenium(III), cluster, alkyl­carboxyl­ate complex, quadruple metal–metal bond, hydrogen bonding

## Abstract

The title compound, [Re_2_(C_3_H_7_COO)_2_Cl_4_{(CH_3_)_2_SO}_2_], comprises binuclear complex mol­ecules [Re—Re = 2.24502 (13) Å] involving *cis*-oriented double carboxyl­ate bridges, four equatorial chloride ions and two weakly bonded O atoms from dimethyl sulfoxide ligands in the axial positions at the Re^III^ atoms. In the crystal, mol­ecules are linked into corrugated layers parallel to (101) by very weak C—H⋯Cl and C—H⋯O hydrogen-bonding inter­actions. C—H⋯Cl hydrogen bonding provides the links between layers to consolidate a three-dimensional framework.

## Abstract 2

A binuclear bis­(carboxyl­ato)dirhenium(III) complex is reported. The compound is a representative of a small class of alkyl­carboxyl­ate complexes involving a quadruple metal–metal bonds

## Chemical context   

Binuclear rhenium(III) clusters are classical complexes with a unique quadruple metal–metal bond (Cotton *et al.*, 2005 ▸, Golichenko & Shtemenko, 2006 ▸). In our previous work we have shown that such compounds with chloride and alkyl­carboxyl­ate equatorial ligands exhibit anti­tumor, anti­radical and hepato- and nephroprotective biological activity with low toxicity (Dimitrov *et al.*, 1978 ▸, Shtemenko *et al.*, 2007 ▸, 2008 ▸, 2009 ▸, 2013 ▸).
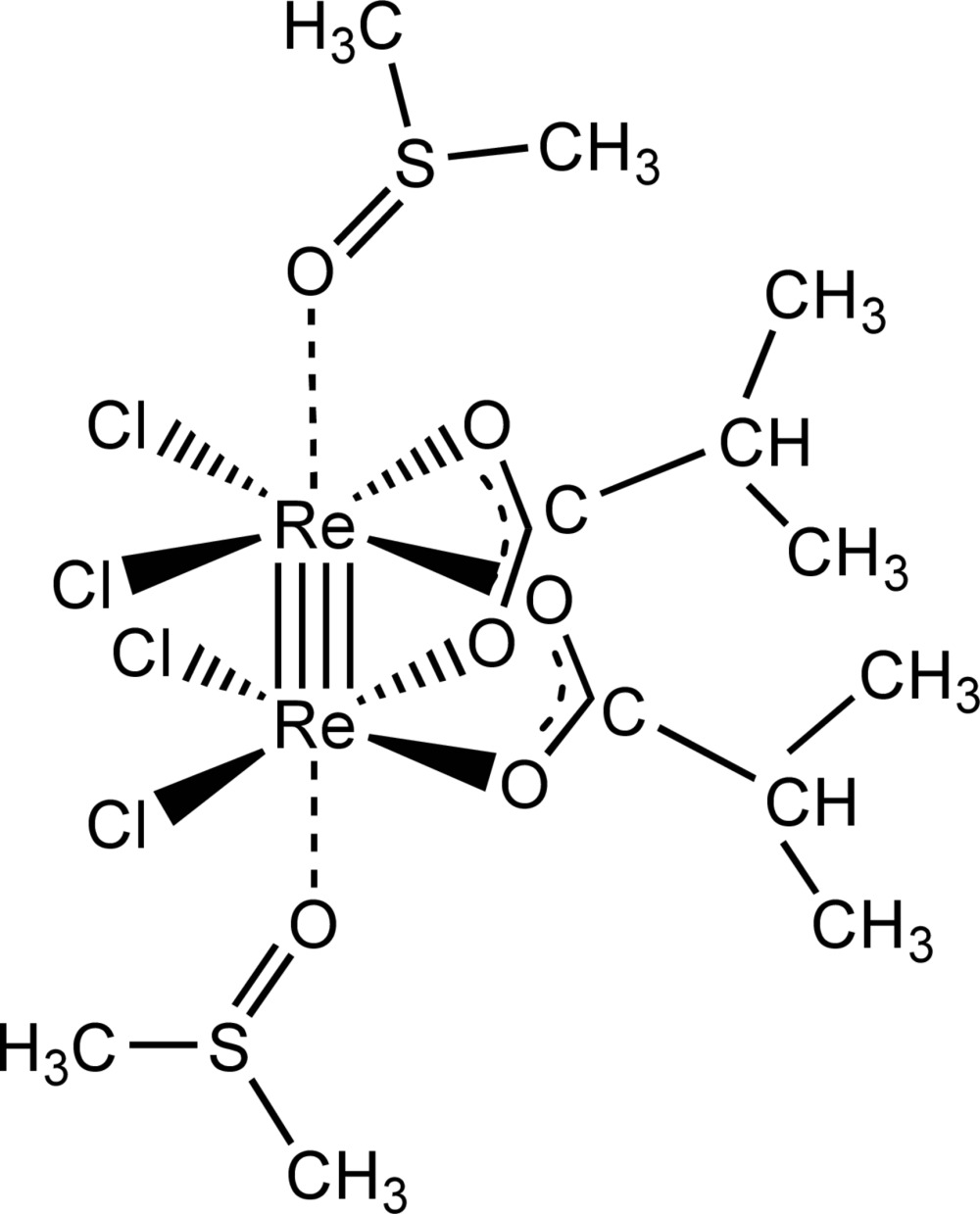


Labile axial ligands and equatorial chloride groups are the reactive centers in inter­actions with other chemical compounds and biological macromolecules *in vitro* and *in vivo* (Shtemenko *et al.*, 2013 ▸). In this context, we present the synthesis and the structure of the title dirhenium(III) complex with isobutyrate equatorial ligands as biologically active groups, which can exhibit anti­tumor activity in the tetra­carboxyl­ate compound Re_2_(*i*-C_3_H_7_COO)_4_Cl_2_ (Shtemenko *et al.*, 2007 ▸).

## Structural commentary   

**Figure 1:**
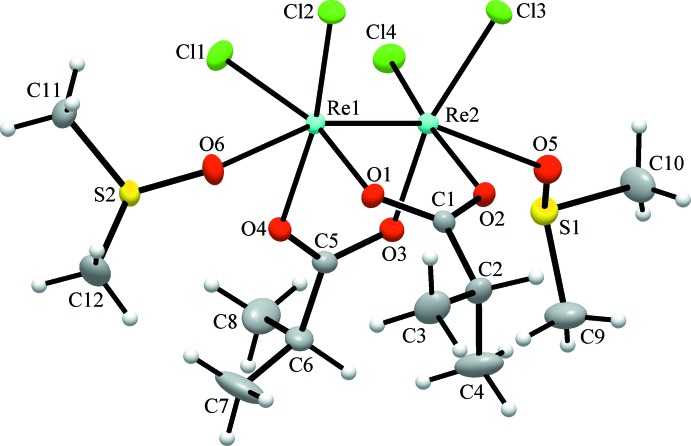
The structure of *cis*-Re_2_Cl_4_{*i*-C_3_H_7_COO}_2_·2(CH_3_)_2_SO, showing displacement ellipsoids drawn at the 50% probability level. H atoms are shown as small spheres of arbitrary radii.

The quadruple Re—Re bond [2.24502 (13) Å] is typical for related di­carboxyl­ato clusters (Cotton *et al.*, 2005 ▸, Shtemenko *et al.*, 2009 ▸) and the coordination of each of the rhenium ions also comprises two chlorides and two oxygen atoms of carboxyl­ate ligands (Fig. 1 ▸). The distorted octa­hedral coordination geometry of Re1 and Re2 is completed by weakly bonded oxygen atoms from dimethyl sulfoxide ligands [Re1—O6 = 2.3282 (15) and Re2—O5 = 2.3938 (15) Å], in *trans*-positions to the Re—Re bond. This may be compared with a similar weak binding of N- or O-donors, which is characteristic of di­carboxyl­atodirhenium compounds (Bera *et al.*, 2003 ▸, Shtemenko *et al.*, 2009 ▸, Golichenko *et al.*, 2015 ▸).

## Supra­molecular features   

**Figure 2:**
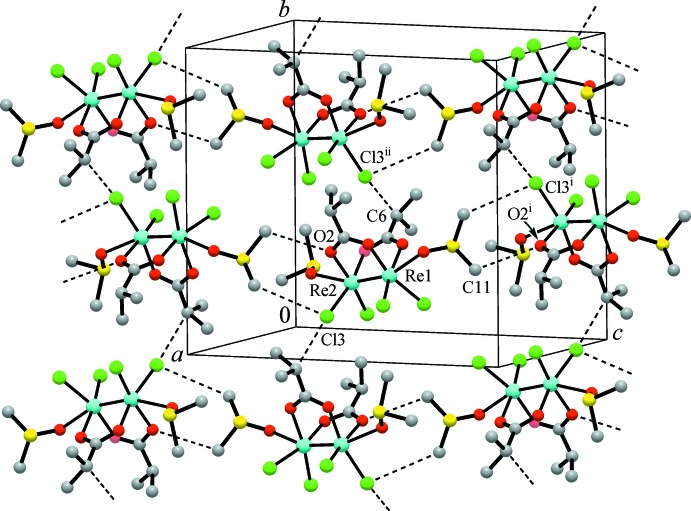
A fragment of the structure, showing weak C—H⋯O and C—H⋯Cl hydrogen-bond inter­actions (dashed lines), which assemble the mol­ecules into corrugated layers parallel to (101). [Symmetry codes: (i) −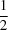 + *x*, 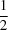 − *y*, 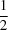 + *z*; (ii) 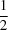 − *x*, 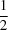 + *y*, 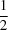 − *z*.]

Inter­molecular bonding is only very weak: it comprises distal, though relatively directional, C—H⋯O and C—H⋯Cl hydrogen-bond inter­actions between the methine- and methyl-H of the carboxyl­ate and DMSO ligands (Table 1 ▸). The shortest bonds found for the chloride acceptors are C6—H6⋯Cl3^ii^ [C6⋯Cl3^ii^ = 3.519 (2) Å; symmetry code (ii): 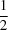 − *x*, 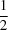 + *y*, 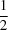 − *z*], which unite the mol­ecules into chains along the *b* axis (Fig. 2 ▸). The hydrogen bonds adopted by two methyl groups of DMSO mol­ecules (referenced by a sulfur atoms S2) assemble these chains into corrugated layers parallel to (101). A very weak bond of this type is found also between adjacent layers: C12⋯Cl2^iii^ = 3.751 (3) Å; symmetry code (iii): −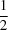 − *x*, 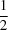 + *y*, 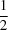 − *z*] (Table 1 ▸). The latter extends the structure into a third direction and provides the formation of a hydrogen-bonded framework.

## Synthesis and crystallization   

[NBu_4_]_2_[Re_2_Cl_8_] (0.2 g, 0.175 mmol) was added to isobutyric acid (10 ml). The mixture was heated for 3 h in a water bath under an inert atmosphere. DMSO (0.5 ml) was then added to the resulting blue solution at room temperature. A dark-blue crystalline product (0.12 g, yield 81%) was obtained after 12 h, was collected by filtration and dried in air.

## Refinement   

Crystal data, data collection and structure refinement details are summarized in Table 2 ▸. All H were refined using a riding-model approximation, with C—H = 0.98–1.00 Å, and with *U*_iso_(H) = 1.2*U*_eq_(C) or 1.5*U*_eq_(C) for methyl H atoms. A rotating model was used for the methyl groups. Six outliers (2 6 1, 3 3 3, 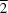 4 3, 0 1 1, 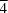 3 4, 3 3 7) were omitted in the last cycles of refinement.

## Supplementary Material

Crystal structure: contains datablock(s) I. DOI: 10.1107/S2056989015017429/rz5165sup1.cif

Structure factors: contains datablock(s) I. DOI: 10.1107/S2056989015017429/rz5165Isup2.hkl

CCDC reference: 1425634

Additional supporting information:  crystallographic information; 3D view; checkCIF report

## Figures and Tables

**Table 1:** Hydrogen-bond geometry (, )

*D*H*A*	*D*H	H*A*	*D* *A*	*D*H*A*
C11H11*B*O2^i^	0.98	2.40	3.324(3)	156
C6H6Cl3^ii^	1.00	2.73	3.519(2)	136
C12H12*A*Cl2^iii^	0.98	2.82	3.751(3)	159
C12H12*B*Cl3^i^	0.98	2.82	3.760(3)	161

Symmetry codes: (i) 
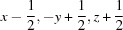
; (ii) 
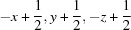
; (iii) 
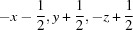
.

**Table 2:** Experimental details

Crystal data	
Chemical formula	[Re_2_(C_4_H_7_O_2_)_2_Cl_4_(C_2_H_6_OS)_2_]
*M*_r_	844.65
Crystal system, space group	Monoclinic, *P*2_1_/*n*
Temperature (K)	110
*a*, *b*, *c* ()	10.5581(4), 14.7406(5), 15.6088(6)
()	100.794(2)
*V* (^3^)	2386.26(15)
*Z*	4
Radiation type	Mo *K*
(mm^1^)	10.78
Crystal size (mm)	0.22 0.18 0.09

Data collection	
Diffractometer	Siemens SMART CCD area-detector
Absorption correction	Multi-scan (*SADABS*; Bruker, 2008 ▸)
*T*_min_, *T*_max_	0.133, 0.478
No. of measured, independent and observed [*I* > 2(*I*)] reflections	93039, 14497, 11921
*R*_int_	0.040
(sin /)_max_ (^1^)	0.909

Refinement	
*R*[*F* ^2^ > 2(*F* ^2^)], *wR*(*F* ^2^), *S*	0.025, 0.049, 1.00
No. of reflections	14497
No. of parameters	243
H-atom treatment	H-atom parameters constrained
_max_, _min_ (e ^3^)	1.71, 1.14

Computer programs: *APEX2* and *SAINT* (Bruker, 2008 ▸), *SHELXS97* (Sheldrick 2008 ▸), *SHELXL2014* (Sheldrick, 2015 ▸), *DIAMOND* (Brandenburg, 1999 ▸) and *WinGX* (Farrugia, 2012 ▸).

## supplementary crystallographic information

### Crystal data

**Table:**

[Re_2_(C_4_H_7_O_2_)_2_Cl_4_(C_2_H_6_OS)_2_]	*F*(000) = 1584
*M**_r_* = 844.65	*D*_x_ = 2.351 Mg m^−^^3^
Monoclinic, *P*2_1_/*n*	Mo *K*α radiation, λ = 0.71073 Å
*a* = 10.5581 (4) Å	Cell parameters from 9919 reflections
*b* = 14.7406 (5) Å	θ = 2.4–39.0°
*c* = 15.6088 (6) Å	µ = 10.78 mm^−^^1^
β = 100.794 (2)°	*T* = 110 K
*V* = 2386.26 (15) Å^3^	Plate, blue
*Z* = 4	0.22 × 0.18 × 0.09 mm

### Data collection

**Table:**

Siemens SMART CCD area-detector diffractometer	11921 reflections with *I* > 2σ(*I*)
Graphite monochromator	*R*_int_ = 0.040
phi and ω scans	θ_max_ = 40.2°, θ_min_ = 2.2°
Absorption correction: multi-scan (*SADABS*; Bruker, 2008)	*h* = −19→18
*T*_min_ = 0.133, *T*_max_ = 0.478	*k* = −25→26
93039 measured reflections	*l* = −28→27
14497 independent reflections	

### Refinement

**Table:**

Refinement on *F*^2^	0 restraints
Least-squares matrix: full	Hydrogen site location: inferred from neighbouring sites
*R*[*F*^2^ > 2σ(*F*^2^)] = 0.025	H-atom parameters constrained
*wR*(*F*^2^) = 0.049	*w* = 1/[σ^2^(*F*_o_^2^) + (0.0183*P*)^2^ + 1.7981*P*] where *P* = (*F*_o_^2^ + 2*F*_c_^2^)/3
*S* = 1.00	(Δ/σ)_max_ = 0.002
14497 reflections	Δρ_max_ = 1.71 e Å^−^^3^
243 parameters	Δρ_min_ = −1.14 e Å^−^^3^

### Special details

**Table:**

Geometry. All e.s.d.'s (except the e.s.d. in the dihedral angle between two l.s. planes) are estimated using the full covariance matrix. The cell e.s.d.'s are taken into account individually in the estimation of e.s.d.'s in distances, angles and torsion angles; correlations between e.s.d.'s in cell parameters are only used when they are defined by crystal symmetry. An approximate (isotropic) treatment of cell e.s.d.'s is used for estimating e.s.d.'s involving l.s. planes.

### Fractional atomic coordinates and isotropic or equivalent isotropic displacement parameters (Å^2^)

**Table:**

	*x*	*y*	*z*	*U*_iso_*/*U*_eq_	
Re1	−0.06723 (2)	0.17363 (2)	0.27880 (2)	0.01130 (2)	
Re2	0.10986 (2)	0.16371 (2)	0.21905 (2)	0.01147 (2)	
Cl1	−0.01954 (5)	0.08982 (4)	0.40554 (3)	0.02107 (9)	
Cl2	−0.19965 (5)	0.05974 (3)	0.21084 (3)	0.01810 (8)	
Cl3	0.06421 (5)	0.04494 (3)	0.12210 (3)	0.01726 (8)	
Cl4	0.25202 (5)	0.07759 (3)	0.31739 (3)	0.02151 (9)	
S1	0.41251 (5)	0.24240 (4)	0.20176 (3)	0.01935 (9)	
S2	−0.23871 (5)	0.25929 (3)	0.42297 (3)	0.01756 (9)	
O1	−0.14493 (13)	0.26371 (9)	0.18365 (9)	0.0142 (2)	
O2	0.02927 (14)	0.25241 (9)	0.12399 (9)	0.0145 (2)	
O3	0.18421 (14)	0.27603 (9)	0.28722 (9)	0.0159 (3)	
O4	0.01121 (14)	0.28533 (9)	0.34802 (9)	0.0158 (3)	
O5	0.28531 (14)	0.20575 (10)	0.14988 (10)	0.0189 (3)	
O6	−0.24413 (14)	0.23612 (10)	0.32675 (9)	0.0189 (3)	
C1	−0.07803 (18)	0.29012 (12)	0.12848 (12)	0.0133 (3)	
C2	−0.1181 (2)	0.37225 (13)	0.07331 (14)	0.0181 (4)	
H2	−0.1090	0.3585	0.0120	0.022*	
C3	−0.2560 (2)	0.40050 (17)	0.07314 (17)	0.0274 (5)	
H3A	−0.2788	0.4516	0.0330	0.041*	
H3B	−0.2647	0.4188	0.1321	0.041*	
H3C	−0.3138	0.3494	0.0542	0.041*	
C4	−0.0227 (3)	0.44808 (16)	0.1087 (2)	0.0354 (6)	
H4A	0.0658	0.4260	0.1125	0.053*	
H4B	−0.0358	0.4662	0.1668	0.053*	
H4C	−0.0371	0.5004	0.0694	0.053*	
C5	0.1204 (2)	0.31512 (13)	0.33854 (12)	0.0152 (3)	
C6	0.1796 (2)	0.39547 (14)	0.38935 (14)	0.0197 (4)	
H6	0.2283	0.4313	0.3518	0.024*	
C7	0.0785 (3)	0.4565 (2)	0.4167 (2)	0.0469 (8)	
H7A	0.0176	0.4764	0.3649	0.070*	
H7B	0.1206	0.5095	0.4476	0.070*	
H7C	0.0321	0.4229	0.4554	0.070*	
C8	0.2750 (3)	0.36103 (19)	0.46883 (17)	0.0337 (6)	
H8A	0.3380	0.3207	0.4495	0.051*	
H8B	0.2282	0.3276	0.5074	0.051*	
H8C	0.3200	0.4127	0.5004	0.051*	
C9	0.4146 (3)	0.36054 (17)	0.1775 (2)	0.0360 (6)	
H9A	0.4047	0.3689	0.1143	0.054*	
H9B	0.4968	0.3868	0.2065	0.054*	
H9C	0.3435	0.3908	0.1984	0.054*	
C10	0.5306 (2)	0.20667 (18)	0.14113 (18)	0.0291 (5)	
H10A	0.5367	0.1403	0.1425	0.044*	
H10B	0.6144	0.2329	0.1669	0.044*	
H10C	0.5057	0.2272	0.0806	0.044*	
C11	−0.3361 (3)	0.17496 (16)	0.46035 (17)	0.0286 (5)	
H11A	−0.4191	0.1709	0.4200	0.043*	
H11B	−0.3508	0.1913	0.5186	0.043*	
H11C	−0.2922	0.1162	0.4631	0.043*	
C12	−0.3441 (3)	0.35344 (17)	0.42132 (18)	0.0338 (6)	
H12A	−0.3087	0.4057	0.3950	0.051*	
H12B	−0.3529	0.3685	0.4811	0.051*	
H12C	−0.4289	0.3381	0.3870	0.051*	

### Atomic displacement parameters (Å^2^)

**Table:**

	*U*^11^	*U*^22^	*U*^33^	*U*^12^	*U*^13^	*U*^23^
Re1	0.01040 (3)	0.01222 (3)	0.01171 (3)	−0.00114 (2)	0.00320 (2)	0.00002 (2)
Re2	0.00953 (3)	0.01154 (3)	0.01360 (3)	0.00081 (2)	0.00288 (2)	0.00041 (2)
Cl1	0.0222 (2)	0.0241 (2)	0.0166 (2)	−0.00111 (18)	0.00305 (17)	0.00674 (16)
Cl2	0.0161 (2)	0.01640 (19)	0.0219 (2)	−0.00426 (15)	0.00380 (17)	−0.00305 (15)
Cl3	0.0169 (2)	0.01521 (19)	0.0201 (2)	0.00138 (15)	0.00460 (16)	−0.00402 (14)
Cl4	0.0167 (2)	0.0215 (2)	0.0244 (2)	0.00463 (17)	−0.00088 (18)	0.00541 (17)
S1	0.0130 (2)	0.0223 (2)	0.0230 (2)	−0.00283 (17)	0.00407 (18)	−0.00136 (17)
S2	0.0194 (2)	0.0189 (2)	0.0162 (2)	−0.00284 (17)	0.00824 (17)	−0.00281 (15)
O1	0.0120 (6)	0.0153 (6)	0.0159 (6)	0.0000 (5)	0.0041 (5)	0.0022 (4)
O2	0.0133 (6)	0.0153 (6)	0.0157 (6)	0.0025 (5)	0.0048 (5)	0.0026 (4)
O3	0.0136 (6)	0.0161 (6)	0.0186 (6)	−0.0023 (5)	0.0044 (5)	−0.0023 (5)
O4	0.0156 (7)	0.0163 (6)	0.0162 (6)	−0.0038 (5)	0.0050 (5)	−0.0034 (5)
O5	0.0108 (6)	0.0225 (7)	0.0238 (7)	−0.0020 (5)	0.0040 (5)	−0.0011 (5)
O6	0.0164 (7)	0.0255 (7)	0.0167 (7)	−0.0009 (5)	0.0076 (5)	−0.0038 (5)
C1	0.0134 (8)	0.0128 (7)	0.0135 (8)	0.0015 (6)	0.0022 (6)	−0.0002 (5)
C2	0.0175 (9)	0.0170 (8)	0.0199 (9)	0.0037 (7)	0.0034 (7)	0.0046 (6)
C3	0.0184 (10)	0.0275 (11)	0.0353 (13)	0.0065 (8)	0.0025 (9)	0.0084 (9)
C4	0.0254 (12)	0.0162 (10)	0.0617 (18)	−0.0007 (8)	0.0004 (12)	0.0048 (10)
C5	0.0161 (9)	0.0132 (8)	0.0155 (8)	−0.0013 (6)	0.0015 (7)	−0.0012 (6)
C6	0.0206 (10)	0.0179 (9)	0.0204 (9)	−0.0062 (7)	0.0028 (7)	−0.0044 (7)
C7	0.0375 (16)	0.0298 (14)	0.072 (2)	−0.0008 (11)	0.0079 (15)	−0.0319 (14)
C8	0.0341 (14)	0.0370 (14)	0.0254 (12)	−0.0122 (11)	−0.0065 (10)	−0.0026 (9)
C9	0.0340 (14)	0.0203 (11)	0.0549 (17)	−0.0051 (10)	0.0113 (13)	−0.0022 (10)
C10	0.0134 (9)	0.0340 (13)	0.0412 (14)	0.0010 (8)	0.0083 (9)	−0.0067 (10)
C11	0.0379 (14)	0.0239 (11)	0.0294 (12)	−0.0072 (9)	0.0206 (11)	−0.0015 (8)
C12	0.0494 (17)	0.0237 (11)	0.0322 (13)	0.0104 (11)	0.0172 (12)	−0.0002 (9)

### Geometric parameters (Å, º)

**Table:**

Re1—O1	2.0459 (13)	C3—H3C	0.9800
Re1—O4	2.0565 (13)	C4—H4A	0.9800
Re1—Re2	2.2450 (1)	C4—H4B	0.9800
Re1—Cl1	2.3065 (5)	C4—H4C	0.9800
Re1—Cl2	2.3115 (5)	C5—C6	1.495 (3)
Re1—O6	2.3282 (15)	C6—C7	1.517 (4)
Re2—O2	2.0401 (13)	C6—C8	1.531 (3)
Re2—O3	2.0437 (14)	C6—H6	1.0000
Re2—Cl3	2.3052 (5)	C7—H7A	0.9800
Re2—Cl4	2.3147 (5)	C7—H7B	0.9800
Re2—O5	2.3938 (15)	C7—H7C	0.9800
S1—O5	1.5310 (15)	C8—H8A	0.9800
S1—C10	1.780 (2)	C8—H8B	0.9800
S1—C9	1.783 (3)	C8—H8C	0.9800
S2—O6	1.5308 (15)	C9—H9A	0.9800
S2—C12	1.776 (3)	C9—H9B	0.9800
S2—C11	1.780 (2)	C9—H9C	0.9800
O1—C1	1.273 (2)	C10—H10A	0.9800
O2—C1	1.276 (2)	C10—H10B	0.9800
O3—C5	1.276 (2)	C10—H10C	0.9800
O4—C5	1.268 (3)	C11—H11A	0.9800
C1—C2	1.500 (3)	C11—H11B	0.9800
C2—C3	1.514 (3)	C11—H11C	0.9800
C2—C4	1.536 (3)	C12—H12A	0.9800
C2—H2	1.0000	C12—H12B	0.9800
C3—H3A	0.9800	C12—H12C	0.9800
C3—H3B	0.9800		
			
O1—Re1—O4	85.93 (6)	C2—C3—H3C	109.5
O1—Re1—Re2	89.58 (4)	H3A—C3—H3C	109.5
O4—Re1—Re2	89.18 (4)	H3B—C3—H3C	109.5
O1—Re1—Cl1	164.51 (4)	C2—C4—H4A	109.5
O4—Re1—Cl1	88.65 (4)	C2—C4—H4B	109.5
Re2—Re1—Cl1	104.857 (14)	H4A—C4—H4B	109.5
O1—Re1—Cl2	90.71 (4)	C2—C4—H4C	109.5
O4—Re1—Cl2	166.43 (4)	H4A—C4—H4C	109.5
Re2—Re1—Cl2	103.960 (13)	H4B—C4—H4C	109.5
Cl1—Re1—Cl2	91.189 (19)	O4—C5—O3	121.00 (17)
O1—Re1—O6	74.92 (5)	O4—C5—C6	120.74 (18)
O4—Re1—O6	77.45 (6)	O3—C5—C6	118.24 (18)
Re2—Re1—O6	160.04 (4)	C5—C6—C7	111.87 (19)
Cl1—Re1—O6	89.75 (4)	C5—C6—C8	108.26 (18)
Cl2—Re1—O6	88.98 (4)	C7—C6—C8	111.1 (2)
O2—Re2—O3	85.80 (6)	C5—C6—H6	108.5
O2—Re2—Re1	89.66 (4)	C7—C6—H6	108.5
O3—Re2—Re1	89.97 (4)	C8—C6—H6	108.5
O2—Re2—Cl3	90.11 (4)	C6—C7—H7A	109.5
O3—Re2—Cl3	165.87 (4)	C6—C7—H7B	109.5
Re1—Re2—Cl3	103.544 (13)	H7A—C7—H7B	109.5
O2—Re2—Cl4	164.60 (4)	C6—C7—H7C	109.5
O3—Re2—Cl4	87.76 (4)	H7A—C7—H7C	109.5
Re1—Re2—Cl4	104.318 (15)	H7B—C7—H7C	109.5
Cl3—Re2—Cl4	92.789 (18)	C6—C8—H8A	109.5
O2—Re2—O5	76.03 (5)	C6—C8—H8B	109.5
O3—Re2—O5	76.76 (5)	H8A—C8—H8B	109.5
Re1—Re2—O5	161.00 (4)	C6—C8—H8C	109.5
Cl3—Re2—O5	89.13 (4)	H8A—C8—H8C	109.5
Cl4—Re2—O5	88.89 (4)	H8B—C8—H8C	109.5
O5—S1—C10	104.28 (10)	S1—C9—H9A	109.5
O5—S1—C9	106.08 (12)	S1—C9—H9B	109.5
C10—S1—C9	97.94 (14)	H9A—C9—H9B	109.5
O6—S2—C12	104.59 (11)	S1—C9—H9C	109.5
O6—S2—C11	104.36 (10)	H9A—C9—H9C	109.5
C12—S2—C11	98.71 (14)	H9B—C9—H9C	109.5
C1—O1—Re1	119.46 (12)	S1—C10—H10A	109.5
C1—O2—Re2	119.54 (12)	S1—C10—H10B	109.5
C5—O3—Re2	119.74 (13)	H10A—C10—H10B	109.5
C5—O4—Re1	120.10 (13)	S1—C10—H10C	109.5
S1—O5—Re2	121.94 (8)	H10A—C10—H10C	109.5
S2—O6—Re1	121.25 (8)	H10B—C10—H10C	109.5
O1—C1—O2	121.00 (17)	S2—C11—H11A	109.5
O1—C1—C2	120.20 (17)	S2—C11—H11B	109.5
O2—C1—C2	118.55 (17)	H11A—C11—H11B	109.5
C1—C2—C3	112.98 (18)	S2—C11—H11C	109.5
C1—C2—C4	106.60 (17)	H11A—C11—H11C	109.5
C3—C2—C4	111.48 (19)	H11B—C11—H11C	109.5
C1—C2—H2	108.6	S2—C12—H12A	109.5
C3—C2—H2	108.6	S2—C12—H12B	109.5
C4—C2—H2	108.6	H12A—C12—H12B	109.5
C2—C3—H3A	109.5	S2—C12—H12C	109.5
C2—C3—H3B	109.5	H12A—C12—H12C	109.5
H3A—C3—H3B	109.5	H12B—C12—H12C	109.5

### Hydrogen-bond geometry (Å, º)

**Table:**

*D*—H···*A*	*D*—H	H···*A*	*D*···*A*	*D*—H···*A*
C11—H11*B*···O2^i^	0.98	2.40	3.324 (3)	156
C6—H6···Cl3^ii^	1.00	2.73	3.519 (2)	136
C12—H12*A*···Cl2^iii^	0.98	2.82	3.751 (3)	159
C12—H12*B*···Cl3^i^	0.98	2.82	3.760 (3)	161

Symmetry codes: (i) *x*−1/2, −*y*+1/2, *z*+1/2; (ii) −*x*+1/2, *y*+1/2, −*z*+1/2; (iii) −*x*−1/2, *y*+1/2, −*z*+1/2.
